# Metagenomic next-generation sequencing: new horizons in microbiology

**DOI:** 10.3389/fcimb.2026.1824160

**Published:** 2026-07-21

**Authors:** Nan Guo, Shihan Chen, Lina Guo, Xiaotong Qiu, Zhenjun Li

**Affiliations:** 1 Research Center for Reverse Microbial Etiology, Workstation of Academician, Shanxi Medical University, Taiyuan, China; 2 School of Public Health, Shanxi Medical University, Taiyuan, Shanxi, China; 3 Key Laboratory of Coal Environmental Pathogenicity and Prevention, Shanxi Medical University, Ministry of Education, Taiyuan, China; 4 School of Clinical Medicine, Southwest Medical University, Luzhou, China; 5 College of International Studies, National University of Defense Technology, Nanjing, Jiangsu, China; 6 National Key Laboratory of Intelligent Tracking and Forecasting for Infectious Diseases, National Institute for Communicable Disease Control and Prevention, Chinese Center for Disease Control and Prevention, Beijing, China

**Keywords:** bioinformatics, clinical diagnostics, metagenomic next-generation sequencing, One Health, pathogen surveillance, public health

## Abstract

The COVID-19 pandemic has exposed vulnerabilities in global health systems while accelerating the adoption of metagenomic next-generation sequencing (mNGS) as a transformative tool for culture-independent, unbiased microbial detection. In clinical diagnostics, mNGS enables simultaneous detection of diverse pathogens without prior hypothesis, though its yield depends heavily on specimen type and clinical context. In public health, mNGS has demonstrated remarkable utility in outbreak tracing, novel pathogen discovery, antimicrobial resistance (AMR) surveillance, and One Health initiatives. However, massive data volumes pose persistent challenges in bioinformatics, standardization, and computational demands. Future integration of artificial intelligence, automated platforms, and multi-omics approaches will enhance the conversion of raw data into actionable insights. Collectively, mNGS is poised to drive a paradigm shift from reactive responses to proactive, system-level microbial surveillance across human, animal, and environmental health.

## 1 Introduction

### 1.1 Background and rationale

The COVID-19 pandemic exposed critical weaknesses in global public health systems, underscoring an urgent need for more resilient and proactive surveillance frameworks—an ambition that hinges fundamentally on innovations in microbial detection technologies. The field of microbial identification has undergone profound transformation, evolving from traditional culture techniques to sophisticated molecular methods. For decades, methods such as culture, microscopy, and biochemical analysis served as the gold standard, forming the cornerstone of microbial testing (Liu et al., 2021). However, these techniques possess inherent limitations, including lengthy detection cycles and low sensitivity toward fastidious or slow-growing bacteria. More fundamentally, their intrinsic bias toward detecting only species cultivable *in vitro* has left vast portions of the microbial world, often termed the “microbial dark matter”, largely unexplored (Rashid and Stingl, 2015). This limitation was overcome by molecular methods, culminating in Metagenomic Next-Generation Sequencing (mNGS)—a hypothesis-free technology that directly sequences all nucleic acids in a sample, enabling comprehensive detection of mixed infections, uncultivable pathogens, and novel microorganisms without prior knowledge of targets (Han et al., 2020; Gauthier et al., 2023). Just as in the early stages of the pandemic, researchers successfully identified a novel bat-derived coronavirus in patients using mNGS, revealing yet another classic case of animal-to-human viral transmission (Wu et al., 2020). In fact, years earlier, Gardy et al. had already highlighted the profound potential of genomic sequencing in identifying, diagnosing, and tracking new pathogens in real-time (Gardy and Loman, 2018). Therefore, mNGS has rapidly emerged as a focal point in microbiology due to its critical role in detecting and tracing unknown pathogens.

The evolution from traditional to molecular methods represents not merely a technological upgrade but a fundamental shift in perspective. Numerous comparative studies have also fully demonstrated the changes brought about by technological innovation. For example, the findings of Wilson et al. on cerebrospinal fluid (CSF) in a selected population showed that mNGS identified more potential pathogens compared to conventional CSF tests, highlighting its significant advantage in diagnosing mixed infections (Wilson et al., 2019). In mainland China, a study by Qu et al. on 299 patients with community-acquired pneumonia from multiple hospitals further demonstrated that the pathogen detection rate of traditional methods (culture, antigen testing, PCR) was 40.8%, which increased to 74.2% when combined with mNGS (Qu et al., 2022), indicating that mNGS can enhance pathogen identification capability. Furthermore, multiple studies on samples including CSF, peritoneal dialysis effluent, blood, bronchoalveolar lavage fluid, and sputum have confirmed that the detection sensitivity of mNGS is higher than that of traditional culture methods (Duan et al., 2021; Liu et al., 2021; Ye et al., 2022; Dong et al., 2023). Other studies comparing mNGS results with culture-based or PCR-based identification further emphasize the advantages of mNGS in detecting unknown pathogens and identifying difficult-to-culture or unculturable microorganisms (Gu et al., 2021; Jia et al., 2023; Yu et al., 2024). Collectively, these studies suggest that mNGS can serve as a powerful supplement or alternative to traditional detection methods. It is particularly important for the rapid and comprehensive identification of unculturable pathogens and mixed infections.

### 1.2 Purpose and scope of the review

This article reviews the role of mNGS as a transformative tool in the field of microbiology, covering a wide range of applications from pathogen detection to supporting “One Health” surveillance. A literature search was conducted using PubMed, with combinations of the terms “metagenomic next-generation sequencing”, “clinical metagenomics”, “mNGS”, “pathogen detection”, “antimicrobial resistance” and “One Health” targeting articles published between January 2000 and May 2026. Priority was given to multicenter evaluations, benchmarking studies, consensus guidelines, and public health implementation reports; additional references were identified through the bibliographies of retrieved articles. Literature screening was guided by relevance to the core research questions rather than by exhaustive coverage of all published studies. We critically evaluate the clinical utility of mNGS across different sample types and explore its applications in public health domains such as outbreak investigation, antimicrobial resistance surveillance, and pathogen discovery, with a focus on the scenarios in which mNGS can provide actionable value. Furthermore, we examine long-standing barriers to the translation of this technology, including standardization, contamination, and the challenge of distinguishing between pathogens and commensal bacteria. We also highlight emerging frontiers such as AI-driven analysis, multi-omics integration, and automated platforms, which hold promise for establishing mNGS as a routine pillar of precision clinical microbiology and proactive public health.

## 2 Metagenomic next-generation sequencing

### 2.1 Concept and definition of mNGS

Metagenomic Next-Generation Sequencing (mNGS), also known as shotgun metagenomic sequencing, enables culture-independent, high-throughput sequencing of all microbial nucleic acids in a sample (Gu et al., 2019). Understanding its distinct role requires a comparative analysis within the broader NGS technology landscape.

Beyond mNGS, two common next-generation sequencing strategies exist: targeted NGS (tNGS) and whole-genome sequencing (WGS) (Rodino and Simner, 2024). Targeted NGS (tNGS), such as 16S rRNA gene sequencing, profiles microbial communities via high-throughput amplification of phylogenetic markers, offering lower costs but limited resolution. In contrast, mNGS sequences all DNA in a sample, enabling more precise species-level identification and functional gene analysis. To compare their performance, the Bars-Cortina team conducted parallel analysis on 156 human fecal samples and found that mNGS outperformed 16S sequencing in both the depth and breadth of microbial community analysis (Bars-Cortina et al., 2024). Durazzi reached similar conclusions in studies on chicken gut microbiota (Durazzi et al., 2021), indicating that mNGS can more comprehensively reveal microbiome complexity. And Ranjan et al. further demonstrated that mNGS provides higher taxonomic resolution, more accurate diversity assessment, and reliable functional gene prediction (Ranjan et al., 2016). These studies collectively demonstrate that mNGS generally provides higher taxonomic resolution and functional gene information than tNGS, making it preferable for strain-level profiling and resistome analysis. However, tNGS remains a cost-effective alternative for large-scale community surveys, especially when host DNA interference is minimal and genus-level identification is sufficient.

Whole-genome sequencing (WGS) performs high-resolution genomic analysis on pure bacterial colonies, deciphering their complete genetic blueprint for precise pathogen typing, virulence and resistance gene identification, and evolutionary studies (Purushothaman et al., 2022). In contrast, mNGS bypasses cultivation, performing unbiased sequencing of DNA from all microorganisms within a sample (Chiu and Miller, 2019). Recognizing the complementary strengths of these approaches, recent studies have begun integrating both technologies. For example, Lu et al. developed a cloud-based open-access CZ ID antimicrobial resistance module that integrates mNGS with single-strain WGS workflows, enabling simultaneous microbial identification and antimicrobial resistance gene profiling (Lu et al., 2025). Similarly, another research team combined WGS and mNGS with machine learning to accurately predict antibiotic resistance in Enterobacteriaceae Complex (ECC), achieving over 95% accuracy in clinical validation while reducing testing time by nearly three days (Pan et al., 2025). These advances demonstrate that integrating WGS precision with mNGS comprehensive coverage is driving pathogen surveillance toward faster, more accurate, and more accessible solutions.

### 2.2 Workflow overview

The mNGS is carried out through a multi-step workflow that combines sophisticated laboratory techniques with advanced computational analysis. The mNGS workflow begins with sample collection and processing, encompassing an extremely wide range of sample types such as stool, blood, cerebrospinal fluid, respiratory secretions, tissues, and environmental samples (Fourgeaud et al., 2024). To ensure analytical validity, this step typically involves pretreatment such as host nucleic acid removal and physical or enzymatic microbial enrichment (He et al., 2022). The extent of host DNA depletion required varies substantially by sample type: an optimized saponin-based protocol, scaled up in reaction volume and combined with adjustments to DNA extraction, whole-genome amplification, and sequencing parameters, enabled efficient pathogen detection in both bloodstream infections and respiratory samples (Kok et al., 2022; Moragues-Solanas et al., 2024). As an alternative strategy, a Benzonase-based approach that pre-digests unprotected DNA from dead cells and host background not only enabled more accurate assessment of the living microbiome in skin samples but also efficiently depleted host reads without distorting microbial community profiles (Amar et al., 2021).

Subsequently, nucleic acid extraction and library construction are performed. This stage requires efficient lysis of various microbial samples using appropriate methods, followed by purification of high-quality total nucleic acids (Zhang et al., 2022). After fragmentation, the resulting DNA fragments undergo end repair and adapter ligation. This is followed by PCR amplification to enrich the library, which is subsequently purified and subjected to quality control using methods such as Qubit quantification and electrophoretic analysis to ensure proper size distribution and purity before final sequencing (Gansauge and Meyer, 2013).

Following library preparation, the process advances to sequencing platform selection. Among numerous platforms (**Table 1**), mainstream second-generation platforms (e.g., Illumina) generate large volumes of short reads with high accuracy and low cost, while third-generation technologies such as Oxford Nanopore Technologies and PacBio yield long-read data, greatly enhancing genome assembly quality and completeness and facilitating the identification of novel species and complex genomic regions (Athanasopoulou et al., 2021; van Dijk et al., 2023; Xia et al., 2023).

**Table 1:** Comparison of mainstream sequencing platforms.

Platform	Core technology	Read length	Throughput & speed	Error profile & accuracy	Key advantages	Main limitations	Typical metagenomics applications	References
Illumina	Sequencing by Synthesis	Short Reads	Very high; Fast (1–3 days)	Substitutions; >99.9% raw accuracy	High accuracy, low cost per Gb; mature protocols	Short reads complicate assembly; GC bias	Species/functional profiling; large cohorts	(Frey et al., 2014; Sevim et al., 2019; Linde et al., 2023)
PacBio	SMRT sequencing	Long Reads	Medium–high; hours to days	Random errors; >99.9% (HiFi mode)	Long accurate reads; detects base modifications; high-quality assemblies	High cost; DNA quantity/quality sensitive	Complete MAGs; structural variant analysis	(Miyamoto et al., 2014; Li et al., 2020; Xie et al., 2020)
Oxford Nanopore	Nanopore sensing	Ultra-long Reads	Flexible; real-time (minutes)	Indels; ~95–98% raw accuracy	Portable; real-time; detects modifications	Higher error rate; compute-intensive basecalling	Field surveillance; complete genomes	(Meslier et al., 2022; Cook et al., 2024; Doorenspleet et al., 2025)

In summary, this comprehensive workflow—spanning from sample preparation to bioinformatics interpretation—enables thorough detection and characterization of microorganisms, transforming complex clinical and environmental samples into actionable genomic information.

### 2.3 Data interpretation and reporting

Data quality control and contaminant management serve as critical first steps in ensuring data reliability, effectively distinguishing genuine biological signals from background noise. Comprehensive quality assessments typically encompass metrics such as sequencing quality scores, GC content, adapter contamination, and read redundancy rates. For instance, Davis et al.’s open-source R package Decontam identifies contaminants through statistical pattern recognition without additional experimental controls and has been widely adopted to reduce technical noise, enabling more precise microbiome analysis (Davis et al., 2018).

However, clean data alone is insufficient. The rapid proliferation of mNGS has generated a data deluge, posing both a challenge and an opportunity: how to integrate and extract biologically meaningful patterns from massive global datasets (Dudhagara et al., 2015; Zhao et al., 2017). We believe that to achieve the leap from data accumulation to knowledge discovery, the key lies in establishing robust computing platforms and analytical tools.

At the platform level, diverse public databases and resources offer varying data coverage, analytical focus, and service models (**Table 2**). For example, the MGnify platform, developed by the European Bioinformatics Institute, exemplifies the evolution toward integrated research infrastructure. Recent advancements include a multifold increase in hosted datasets, a non-redundant protein database exceeding 2.4 billion sequences, and a relational database linking sequence annotations to original genomes and sample context (Mitchell et al., 2020). Version 5.0 introduced standardized, reproducible pipelines with support for long-read sequences, ITS region classification, deep learning-assisted annotation, and metabolic pathway prediction. Enhanced APIs, Jupyter Lab integration, and improved visualization tools further support an interactive, end-to-end analytical ecosystem (Richardson et al., 2023). MGnify’s trajectory reflects a broader shift: metagenomic platforms are evolving from passive data repositories into comprehensive infrastructures integrating standardized workflows, interactive analysis, and scalable computing. We note that, although public databases are indispensable, many specialized laboratories still rely on internally curated sequence collections, and both BLAST and BWA alignment technologies are widely used, with the latter being known for its superior performance (Makino et al., 2024).

**Table 2:** Comparison of several metagenomic databases.

Database	Type & scope	Data volume & sources	Analysis features	Advantages	Limitations	References
MGnify	Integrated resource + analysis	>500k samples; diverse environments	Standardized workflows; visualization	One-stop platform; scalable; open access	Data heterogeneity; reference dependence	(Gurbich et al., 2023)
MAGdb	Curated MAG repository	99,672 MAGs from 13.7k samples	Quality-controlled; GTDB-based taxonomy	High-quality data; uniform annotations	Clinical sample bias	(Ye et al., 2025)
CARD	Antibiotic resistance database	>3k curated ARO terms; >15k pathogen isolates	RGI/AMRFinderPlus integration; ontology	Expert-curated; validated; tool interoperability	Narrow focus on AMR	(Jia et al., 2017; Alcock et al., 2020; Mahfouz et al., 2020; Alcock et al., 2023)
GTDB	Genome-based taxonomy	>400k bacterial/archaeal genomes	GTDB-Tk for classification	Phylogenetically consistent; standardized ranks	Framework only; evolving taxonomy	(Chaumeil et al., 2019; Parks et al., 2020; Parks et al., 2022)
UniRef	Non-redundant protein clusters	Comprehensive protein sequence coverage	Functional annotation (KEGG, COG)	Broad functional coverage; well-established	No direct taxonomy	(Suzek et al., 2007; Suzek et al., 2015; Mirdita et al., 2017)

At the tool level, numerous specialized software packages now support multidimensional data interpretation (**Table 3**). For example, SURPI+ integrates taxonomic classification, filtering, and pathogen detection algorithms (Miller et al., 2019)—in one study, this tool identified 797 microbial species in cerebrospinal fluid samples, including DNA viruses, RNA viruses, bacteria, fungi, and parasites (Benoit et al., 2024). However, no single tool is universally suitable, and selection depends heavily on specific research objectives (Ye et al., 2019). Despite progress, major limitations persist. Two rounds of the Critical Assessment of Metagenome Interpretation (CAMI) challenge revealed that existing tools perform well at higher taxonomic levels and in simple communities but struggle with real-world sample heterogeneity—particularly in achieving strain-level resolution, accurately identifying viruses and archaea, and ensuring reproducibility (Sczyrba et al., 2017; Meyer et al., 2022). Thus, while the toolbox for metagenomic analysis continues to expand, realizing its full potential depends on overcoming these persistent limitations through improved algorithms and rigorous performance evaluation.

**Table 3:** Comparison of core metagenomic data analysis software.

Tool	Stage	Function	Input	Advantages	Limitations	References
FastQC	Quality control	Raw read quality reporting	FASTQ	Standard; intuitive	No processing	(Bittencourt, 2010; Wingett and Andrews, 2018)
MultiQC	QC aggregation	Summarizes multiple reports	Tool outputs	Project-level overview; efficient	Dependent on input	(Ewels et al., 2016)
Trimmomatic	Preprocessing	Adapter & quality filtering	FASTQ	Flexible; widely used	Manual parameter tuning	(Bolger et al., 2014; Maran and Davis, 2022; Tu et al., 2024)
KneadData	Preprocessing	Host sequence removal	FASTQ	Optimized for metagenomics	Requires host genome	(Gao et al., 2025)
Kraken2	Taxonomy	k-mer-based classification	FASTQ/contigs	Fast; low memory usage; high detection sensitivity	High false-positive rate; relative abundance	(Wood et al., 2019; Terron-Camero et al., 2022; Rumbavicius et al., 2023)
MetaPhlAn	Taxonomy	Marker-based profiling	FASTQ	Species-level precision; efficient	Database-dependent	(Segata et al., 2012; Blanco-Miguez et al., 2023; Manghi et al., 2023)
MEGAHIT	Assembly	*De novo* metagenomic assembly	FASTQ	Memory-efficient; scalable	Lower contiguity in complex samples	(Li et al., 2015; Li et al., 2016; Wang et al., 2020)
metaSPAdes	Assembly	*De novo* assembly	FASTQ	High contiguity	Resource-heavy	(Nurk et al., 2017; Fritz et al., 2019; Allio et al., 2020)
MetaBAT2	Binning	Genome reconstruction from contigs	Contigs + BAM	Reliable single tool	Benefits from bin integration	(Yue et al., 2020; Mori et al., 2023)
MetaWRAP	Binning	Pipeline with bin refinement	Contigs + BAM	Automated; improves bin quality	Computationally demanding	(Uritskiy et al., 2018)
HUMAnN	Functional	Gene family & pathway quantification	FASTQ	Taxon-stratified output; comprehensive	Complex output	(Beghini et al., 2021)
Prokka	Annotation	Rapid genome annotation	Genomes (MAGs)	Fast; integrated databases	Prokaryote-focused	(Seemann, 2014; Karimi et al., 2021)

In summary, mNGS achieves comprehensive analysis of microbial communities in complex samples by integrating standardized wet-lab procedures with advanced bioinformatics analysis. With ongoing optimization of computational platforms and continuous refinement of analytical tools, mNGS is increasingly becoming a widely adopted research method among scientists.

## 3 Applications of mNGS in clinical diagnosis

Metagenomic next-generation sequencing (mNGS) has evolved from a research-focused tool into a transformative approach in clinical diagnostics, offering the ability to detect bacterial, viral, fungal, and parasitic pathogens simultaneously, without prior hypothesis or culture requirements (Rodino and Simner, 2024). Its clinical adoption, however, is nuanced: the diagnostic yield of mNGS is highly dependent on specimen type, patient context, and pathogen biology, highlighting the importance of tailored application rather than universal deployment (Filkins et al., 2020). The following sections examine these applications across the most common clinical specimens.

### 3.1 Blood

Blood, despite being one of the most accessible clinical specimens, presents unique challenges for mNGS due to the overwhelming background of host DNA relative to microbial content. To overcome this, two complementary strategies have been developed: direct metagenomic sequencing with host DNA depletion, and the analysis of circulating microbial cell-free DNA (cfDNA). cfDNA-based approaches have demonstrated particular promise, detecting pathogens with high sensitivity and identifying infections even in culture-negative patients (O'Grady, 2019; Kanaujia et al., 2023; Hu et al., 2025). For example, cfDNA mNGS detected causative organisms in over 30% of cases where traditional blood culture failed (Blauwkamp et al., 2019; Zhang et al., 2022), and serial cfDNA measurements provided insight into infection dynamics, such as differentiating definite from possible device-related endocarditis (Karchmer et al., 2025). Mechanistic studies have further clarified that cfDNA levels during sepsis primarily reflect impaired hepatic clearance rather than excessive cell death, and nucleosome footprints retained in cfDNA can trace tissue origin, offering both diagnostic and pathophysiological insights (Balks et al., 2025; Cano-Gamez et al., 2025). Notably, combining cfDNA and cellular DNA mNGS enhances overall accuracy, reflecting the complementary nature of these approaches (Chen et al., 2024). Advances in workflow and platform design, such as extraction-free library preparation, have substantially increased microbial read recovery while reducing costs, moving blood-based mNGS closer to routine clinical feasibility (Babb et al., 2025). In sum, cfDNA-based NGS has transformed blood from a challenging matrix into a diagnostically actionable specimen, although the interpretation of these results must be tailored to the specific clinical context.

### 3.2 Bronchoalveolar lavage fluid

BALF provides a direct window into the lower respiratory tract, making it an informative specimen for pneumonia and other pulmonary infections. mNGS of BALF consistently demonstrates superior sensitivity compared with conventional culture, particularly for fungi and opportunistic pathogens (Chen et al., 2022; Huang et al., 2024). In immunocompromised patients, BALF mNGS not only improves diagnostic accuracy but also informs treatment strategies that enhance clinical outcomes (Zhou et al., 2025). Importantly, randomized clinical trials have confirmed that integrating mNGS with conventional microbiological testing can shorten time to clinical improvement in severe pneumonia (Wu et al., 2025). Targeted NGS approaches now offer cost-effective alternatives, though their narrower scope may miss certain pathogens, underscoring the continued value of comprehensive metagenomics (Yin et al., 2024). Future diagnostic advances are likely to arise from integrating pathogen detection with host-response profiling, resolving the long-standing challenge of distinguishing colonization from true infection (Tang et al., 2025a).

### 3.3 Cerebrospinal fluid

CSF represents a high-value matrix for mNGS due to the inherent sterility of the central nervous system (CNS), which simplifies interpretation: any detected microbial DNA or RNA is suspicious. Nonetheless, the low biomass of CSF increases susceptibility to contamination. Clinical studies have consistently shown that mNGS outperforms conventional diagnostics for CNS infections, identifying pathogens that would otherwise remain undetected and enabling rapid, life-saving interventions (Piantadosi et al., 2021; Benoit et al., 2024; Ferreira et al., 2025). Hybrid capture and methylated DNA depletion have expanded the detectable pathogen spectrum (Piantadosi et al., 2021), while integration with host transcriptomic data and machine learning models has enhanced sensitivity and specificity for challenging diagnoses such as tuberculous meningitis (Ramachandran et al., 2022). CSF mNGS thus represents both a diagnostic and discovery tool, uncovering emerging pathogens while redefining the boundaries of CNS infectious disease detection.

### 3.4 Other sample types

Beyond blood, BALF, and CSF, mNGS has demonstrated utility across tissue biopsies, urine, synovial fluid, pleural fluid, and other sterile body fluids. For tissue samples, mNGS can detect rare or fastidious pathogens that conventional methods miss, directly influencing clinical management in nearly half of tested cases (Tang et al., 2025b). In urinary tract infections, rapid targeted and metagenomic approaches outperform culture in both speed and sensitivity, detecting viral and polymicrobial infections that conventional methods overlook (Wang et al., 2023; Chang et al., 2025). Synovial fluid mNGS has been used to optimize preoperative culture strategies, increasing diagnostic yield (Fang et al., 2021). However, the clinical impact of mNGS remains constrained by operational factors such as turnaround time, which can limit timely therapeutic decision-making (Bay et al., 2026). These findings emphasize that specimen selection, context-aware interpretation, and integration with conventional diagnostics are essential to maximize the clinical value of mNGS.

**Figure 1:**
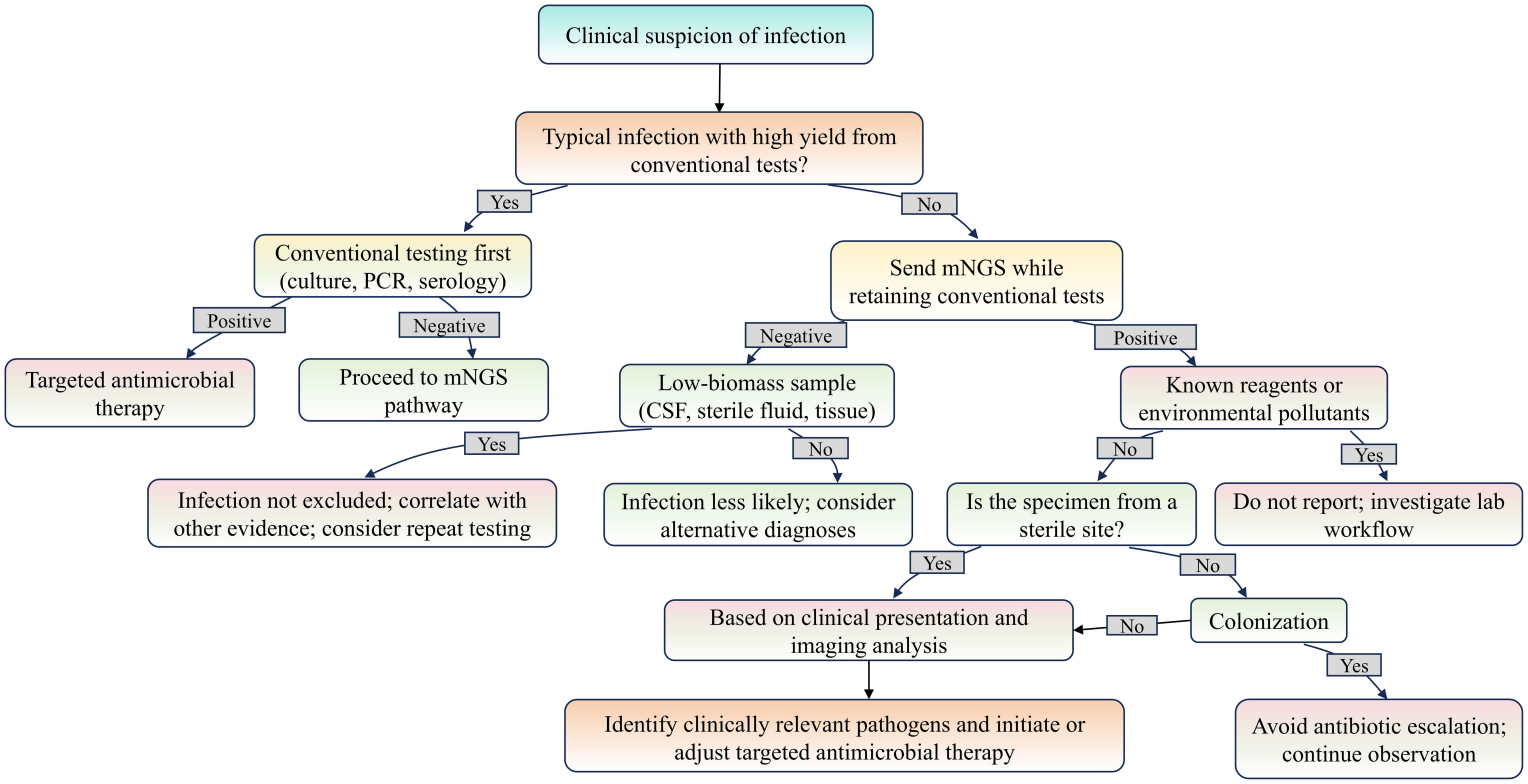
The decision-making framework for the diagnosis of clinical infections.

The above studies indicate that the clinical value of mNGS is not fixed but depends heavily on three interacting factors: specimen type, the host’s immune status, and the clinical question being addressed. Therefore, we have developed a decision-making framework to help clinicians make more informed diagnostic decisions (**Figure 1**).

## 4 Applications of mNGS in public health surveillance

Driven by falling costs and rising automation, high-throughput sequencing is transforming pathogen genomics and public health surveillance (Nicholson et al., 2025)—shifting the paradigm from reactive detection to proactive, data-driven prediction and precision response across critical domains, including pathogen discovery, outbreak investigation, antimicrobial resistance tracking, and One Health surveillance.

### 4.1 Microbial dark matter discovery

The vast diversity of undiscovered microorganisms represents most of Earth’s biodiversity and genetic resources. mNGS now offers an unprecedented technological path for exploring this “microbial dark matter” and deciphering the structure and function of the global microbiome.

In expanding the boundaries of microbial diversity knowledge, Nayfach et al. reconstructed >50,000 high-quality MAGs from >10,000 global samples, >70% of which were novel species (Nayfach et al., 2021). Parks et al. added 7,903 bacterial and archaeal genomes from public datasets, expanding phylogenetic diversity by >30% and identifying 20 candidate phyla (Parks et al., 2017). For RNA viruses—largely undetectable by traditional methods—metagenomics coupled with a deep learning algorithm (LucaProt) recently identified 161,979 potential novel species and 180 viral groups across global ecosystems (Hou et al., 2024).

mNGS has also demonstrated exceptional value in novel pathogen discovery. During the COVID-19 pandemic, Chen et al. used mNGS on bronchoalveolar lavage fluid from two critically ill patients to rapidly identify a novel coronavirus closely related to bat coronaviruses, swiftly pinpointing the outbreak source and alerting global health systems (Chen et al., 2020). Similarly, Crawford et al. applied mNGS to identify a novel Pseudomonas species from a patient with transfusion-associated sepsis, tracing it back to contaminated platelet products (Crawford et al., 2020).

These studies highlight the revolutionary capability of mNGS to uncover previously undetectable microbial taxa, illuminating the “microbial dark matter.” In the coming years, mNGS will drive a new wave of pathogen discovery and solidify its foundational role in global public health defense.

### 4.2 Infectious disease prevention and control

mNGS empowers infectious disease prevention and control through high-resolution pathogen typing, precise transmission chain tracking, phenotypic inference, and informed vaccine and drug design—providing a critical foundation for precision public health strategies (Maljkovic Berry et al., 2020).

For instance, Manara et al. sequenced 184 Staphylococcus aureus isolates, revealing 80 distinct lineages—including five novel clones—and demonstrating how NGS can uncover clinically relevant diversity to inform infection control and treatment (Manara et al., 2018). Beyond fine typing of known pathogens, genomic technology has also proven decisive in outbreak tracing. In December 2020, when a cluster of severe community-acquired pneumonia of unknown cause occurred in a Shandong hospital, mNGS analysis of 12 confirmed cases revealed that six were employees of the same duck meat processing plant. This clue led to further testing of 61 close contacts, ultimately confirming Chlamydia psittaci as the causative agent—a striking demonstration of mNGS’s capacity for rapid pathogen identification and epidemiological tracing during outbreaks of unknown etiology (Zhang et al., 2022).

Extending these applications, Robinson et al. systematically outlined analytical methods for metagenomic data to discover novel biosynthetic enzymes, integrating computational prediction with experimental validation to mine natural product gene clusters and explore protein function (Robinson et al., 2021). This work provides a roadmap for linking gene sequences to protein phenotypes. Genomics also directly informs vaccine development: during the 2014–2015 influenza season in the U.S., genomic sequencing revealed that reduced vaccine effectiveness was associated with hemagglutinin (HA) gene variations in influenza A(H3N2) viruses (Flannery et al., 2016).

Together, these studies illustrate that mNGS—from micro-level genotyping and enzyme discovery to macro-level source tracing—has become an indispensable tool in epidemiological prevention and control.

### 4.3 AMR surveillance

Antimicrobial resistance (AMR), driven largely by antimicrobial misuse, is a leading global health threat (Waddington et al., 2022). Containing its spread requires understanding the complex interactions between pathogens, resistance genes (ARGs), and mobile genetic elements across the human–animal–environment interface—a challenge for which mNGS offers new analytical solutions (Wheeler et al., 2023).

The scale of this threat is alarming. A meta-analysis encompassing 274 studies revealed that 35% of hematologic malignancy patients globally are affected by AMR, with significantly increased mortality risk, and substantial data gaps persist in low- and middle-income regions (Sallah et al., 2025). In clinical settings, Serpa et al. demonstrated that mNGS not only predicts resistance in lower respiratory tract infections but also reveals ARG accumulation and transmission risks within hospital environments—offering a technical pathway for rapid, precise monitoring when combined with Cas9 enrichment and nanopore sequencing (Serpa et al., 2022).

Beyond healthcare, animal husbandry is a major driver of AMR spread. Peng et al. found multidrug resistance in 91% of E. coli isolates from pig farms across mainland China (Peng et al., 2022), while Baker et al. identified 145 potentially mobile ARGs common to chickens and their environment across ten poultry farms, including multiple clinically relevant genes (Baker et al., 2023). These findings underscore that AMR has crossed species barriers, posing zoonotic transmission risks.

The environment mirrors this crisis. Zhao et al. analyzed nearly 4,000 metagenomic datasets across 12 habitats, revealing increasing ARG risks in soil and growing connectivity to the human resistome (Zhao et al., 2025). In sewage, Hendriksen et al. examined 79 sites across 60 countries, finding that ARG diversity and abundance reflect local socioeconomic and sanitary conditions—underscoring sanitation as key to controlling global spread (Hendriksen et al., 2019). Munk et al. further showed that 49 common ARGs exhibit highly diverse, often plasmid-borne genetic contexts, indicating multiple evolutionary origins and the need for locally tailored interventions (Munk et al., 2022). In hospital wastewater, Chen et al. detected 1,237 ARG subtypes, with 94 of 135 plasmid elements mobile—highlighting clear targets for interruption (Chen et al., 2025).

However, inferring AMR from metagenomic data carries several important limitations that must be acknowledged to avoid overinterpretation. First, the detection of an ARG does not consistently predict phenotypic resistance; the presence of a gene does not guarantee its expression, and silent or truncated variants may be counted as functional determinants by standard annotation pipelines (Boolchandani et al., 2019). Second, short-read sequencing often fails to resolve the genomic context of detected ARGs—specifically, whether a resistance gene is located on the chromosome, a plasmid, or a mobile element, and to which pathogen it belongs—information that is critical for assessing transmission risk (Sydenham et al., 2019; Tamma et al., 2019). Third, the choice of database and abundance normalization method can substantially alter the resistome profile generated from the same metagenomic dataset (Mahfouz et al., 2020). Fourth, distinguishing between intrinsic resistance, acquired resistance, and housekeeping gene variants that do not confer clinically relevant resistance is essential for accurate risk assessment but remains challenging with current bioinformatic workflows (McArthur and Wright, 2015). Finally, a meaningful AMR risk assessment should integrate not only gene presence but also mobility potential, host range, pathogenic host association, gene context, and clinical relevance—a level of analysis that current metagenomic pipelines only partially achieve.

Together, these studies demonstrate mNGS’s irreplaceable role in AMR surveillance. By revealing the distribution, flow, and evolution of resistance at the genetic level, mNGS provides the molecular epidemiological evidence essential for understanding transmission mechanisms, assessing regional risks, and formulating precise intervention strategies.

### 4.4 One health framework

The One Health concept emphasizes the interdependence of human, animal, and environmental, while mNGS offers an unprecedented unified perspective for understanding and addressing complex health threats by simultaneously monitoring multidimensional microbial communities (Bertram et al., 2024).

At the foundational level, large-scale gene catalogs established by mNGS—such as the 3.3 million non-redundant gut microbial genes cataloged by Beijing Genomics Institute (BGI) (Qin et al., 2010) and the functional profiling of multiple body sites by the Human Microbiome Project (Human Microbiome Project, C, 2012) —provide essential resources for linking microbiomes to human health.

In environmental monitoring, mNGS has revealed key drivers of microbial distribution. Xiong et al. demonstrated that human activities disrupt protozoa–bacteria trophic structures, offering mechanistic insights into ecosystem decline (Xiong et al., 2021). Sunagawa et al. mapped global ocean microbiomes, identifying temperature as a primary compositional driver (Sunagawa et al., 2015). Extending this, Liddicoat et al. quantitatively linked soil microbiome function to human health, providing tools for environment-based health interventions (Liddicoat et al., 2024).

Critically, mNGS enables precise tracking of pathogens across species. Shi et al. discovered extensive novel viruses and bacteria in farmed and wild mammals, revealing cross-species transmission risks (Shi et al., 2025). Another study identified a rodent-derived α-coronavirus capable of infecting humans, highlighting the need for broader surveillance beyond β-coronaviruses (Park et al., 2025). Most conclusively, Liu et al. provided direct evidence of pseudorabies virus transmission from pigs to humans causing severe encephalitis, confirming significant zoonotic risk (Liu et al., 2021).

In food safety, Carlino et al. built a large-scale food metagenome database, confirming strain-level transmission of food microbes to the human gut (Carlino et al., 2024). Lee et al. employed nanopore sequencing technology to reveal distinct microbial communities and antibiotic resistance gene profiles across different food types, deepening understanding of risks throughout the entire “farm-to-table” chain (Lee et al., 2024).

Despite these advances, translating One Health metagenomic surveillance from proof-of-concept studies into routine practice faces substantial implementation barriers. Harmonized sampling protocols across human, animal, and environmental domains remain underdeveloped, introducing systematic variability that complicates cross-study comparisons (Zinsstag et al., 2020). Metadata standards are inconsistent, with critical contextual information—host demographics, antibiotic exposure, geospatial data—frequently absent or recorded in incompatible formats. Cross-sector governance structures are nascent: effective One Health surveillance requires coordination among public health, veterinary, environmental, and food safety agencies that often operate under separate mandates with limited data-sharing mechanisms (Zinsstag et al., 2023). Privacy and data sovereignty concerns are particularly acute when genomic data cross-national borders, and mandatory data-sharing requirements risk perpetuating inequities where samples from low- and middle-income countries are analyzed in high-income settings without equitable benefit-sharing (Cleaveland et al., 2017). Laboratory comparability remains a critical gap, as significant interlaboratory variability in mNGS performance is magnified when comparing workflows across clinical, veterinary, and environmental laboratories operating under different quality assurance standards (Mogeni et al., 2021). Finally, a fundamental gap persists between detecting a metagenomic signal and mounting an actionable intervention; the evidentiary standards, decision-making frameworks, and communication pathways that would enable such translation are not yet established (Li et al., 2025). Closing this gap will require sustained investment in governance infrastructure, workforce development, and equitable international partnerships alongside continued technical innovation.

In summary, mNGS serves as a core enabling technology within the One Health framework, linking human, animal, and environmental microbiomes to provide the integrated evidence base needed for sustainable health ecosystems.

## 5 Challenges and future perspectives

### 5.1 Challenges and limitations

Although mNGS has been widely adopted, it still faces numerous challenges, including the risk of contamination in samples with low biomass, the effective removal of host DNA, and the need to accurately distinguish between pathogens, commensal bacteria, and background contaminants. To tackle reagent contamination in low-input experiments, researchers developed a statistical method that quantifies contaminants by establishing an inverse relationship between read depth and sample quality—enabling genuine pathogen signal detection without external controls or prior knowledge of contaminants (Zinter et al., 2019). However, contamination remains a common issue for samples with low biomass, such as cerebrospinal fluid; strategies that include DNase treatment to enrich microbial material, combined with bioinformatics tools such as Kraken2 for classification, can improve the reliability of mNGS results (Wang et al., 2026). As a complementary solution to host DNA interference, nanopore adaptive sampling (NAS) technology enables real-time rejection of host sequences as molecules pass through the pore, increasing the microbial sequencing yield of clinical bronchoalveolar lavage fluid samples by eight-fold (Cheng et al., 2022). In terms of genome assembly, an evaluation of 40 sequencing and computational strategies identified hybrid assembly (short plus long reads) combined with metaHiC-based classification as the optimal approach for obtaining high-quality metagenome-assembled genomes (Jia et al., 2023). Beyond detection, Langelier et al. developed a composite diagnostic model integrating pathogen, microbiome, and host response data for lower respiratory tract infections, achieving 100% negative predictive value and demonstrating its capacity to exclude non-infectious cases (Langelier et al., 2018). Separately, New et al. highlighted that functional annotation databases for environmental microbes lag far behind those for model organisms, posing significant bottlenecks for metabolic assembly and classification in heterogeneous samples (New and Brito, 2020). Overall, while mNGS has revolutionized microbial research, technical challenges continue to constrain its potential—making their resolution a critical research priority.

Furthermore, clinical relevance validation and reproducibility require urgent improvement. A large-scale interlaboratory study evaluating mNGS protocols across 90 laboratories revealed significant accuracy and comparability issues in clinical translation (Han et al., 2022). This underscores the need to enhance detection accuracy in low-biomass samples, microbial identification, and quantitative analysis. Standardizing workflows and establishing rigorous quality control systems are therefore critical for ensuring reliable clinical application.

Efforts toward this goal are underway. Zhang et al., for instance, addressed the lack of standardized protocols for nanopore metagenomic sequencing in pneumonia diagnosis by optimizing key parameters—including cell wall lysis, fragment selection, host DNA removal, and sequencing depth—establishing a validated workflow (Zhang et al., 2025). Such practices demonstrate that systematic optimization and standardization can effectively address clinical bottlenecks, driving mNGS from a research tool toward a reliable diagnostic platform.

Economic and logistical barriers are equally significant. High costs, shortages of specialized personnel, and substantial computational demands limit mNGS adoption in resource-constrained settings. Greninger argued that while mNGS holds promise as a disruptive diagnostic tool, it is unlikely to become a universal “one test to rule them all” in the near term; given its cost and limitations, it should currently be positioned as a “final diagnostic” tool for resolving challenging cases (Greninger, 2018). Reducing costs, improving infrastructure, and training specialized personnel are therefore essential for broader clinical application.

In resource-limited regions, identifying pathogens is critical for assessing disease burden and setting surveillance priorities. Studies in Cambodia and Ethiopia have used mNGS to investigate zoonotic transmission and infant health, with findings expected to inform national surveillance programs (Bohl et al., 2022; Mekuria et al., 2025). Accelerating the deployment of high-throughput sequencing in underdeveloped areas will be key to improving local public health outcomes.

Furthermore, ethical and legal concerns—particularly regarding data privacy and equitable access—are becoming increasingly prominent, driving innovations in both technical and policy frameworks. For privacy, tools like COLLAGENE enable secure, collaborative genomic analysis through encryption, allowing data sharing without exposing raw sensitive information (Li et al., 2023). On equity, however, Evertsz et al. caution that mandatory data-sharing requirements may inadvertently exacerbate inequalities and risk perpetuating neocolonial dynamics in global health research (Evertsz et al., 2023). Achieving genuine equity, therefore, requires not only best practices but also fundamental reforms to address structural imbalances within the research system.

In short, addressing these challenges requires a concerted effort involving technological optimization, guideline development, resource investment, and international collaboration.

### 5.2 Future perspectives and innovations

In recent years, artificial intelligence (AI) has emerged as a key driver of multidisciplinary progress. In the life sciences, AI enables efficient processing and integration of multidimensional omics data—including metagenomics, transcriptomics, and proteomics—addressing challenges posed by the high dimensionality and complexity of such datasets (Wu et al., 2024). With its powerful pattern recognition capabilities, AI is fundamentally reshaping genome-resolved metagenomics (Qayyum et al., 2025).

Concrete examples abound. Ruffolo et al. used AI to mine genomic data and design OpenCRISPR-1, a novel high-performance gene editor (Ruffolo et al., 2025). In disease research, machine learning methods identified gut microbial signatures distinguishing metastatic from non-metastatic pancreatic cancer, offering potential intervention targets (Villani et al., 2024). In AMR surveillance, deep learning has enabled large-scale discovery of novel antimicrobial peptide sequences from human gut and environmental microbiomes (Ma et al., 2022; Santos-Junior et al., 2024). In public health, AI can integrate heterogeneous data to enable early pathogen warning, accelerate transmission tracing, and inform countermeasure development—shifting pandemic response from reactive to proactive (Chiu et al., 2023). In summary, the vast data generated by metagenomic sequencing provides a foundation for AI to transform raw information into biological insights and intervention strategies across domains ranging from gene editing to pandemic early warning.

Additionally, high automation and integration represent a major direction for mNGS development, streamlining workflows such as library preparation and accelerating the decentralization of clinical sequencing to frontline healthcare settings. During the pandemic, Rachiglio et al. demonstrated this potential using the fully automated Genexus Integrated Sequencer, obtaining complete SARS-CoV-2 genomes from 21 of 27 clinical samples—including a 40% recovery rate even in low-viral-load specimens (Rachiglio et al., 2021).

Beyond automation, multi-omics integration is increasingly essential. Integrating proteomics, metabolomics, and transcriptomics from the same samples has yielded insights across fields—from molecular signatures of severe malaria (Sobota et al., 2025) and immune function in HIV (Botey-Bataller et al., 2025) to therapeutic targets in breast cancer (Jiang et al., 2024). These studies collectively demonstrate that the development of automated platforms and multi-omics integration analysis is driving innovation in mNGS, providing more powerful tools and deeper insights for precision medicine.

Looking ahead, continuous improvement of databases, analytical algorithms, and AI-driven data mining will enable more systematic exploration of microbial community dynamics, functional gene distribution, and evolutionary relationships. This progress promises to maximize data value extraction across environmental monitoring, pathogen surveillance, biomarker discovery, and functional element mining—propelling metagenomics from large-scale data accumulation toward mechanistic discovery and translational application.

## 6 Conclusion

In summary, mNGS offers three key advantages over traditional methods: it enables broad, hypothesis-free detection of microorganisms, links species identity to functional potential, and allows systematic exploration of the previously inaccessible microbial world—transforming microbiological research from fragmented speculation into a holistic, unbiased science.

**Figure 2:**
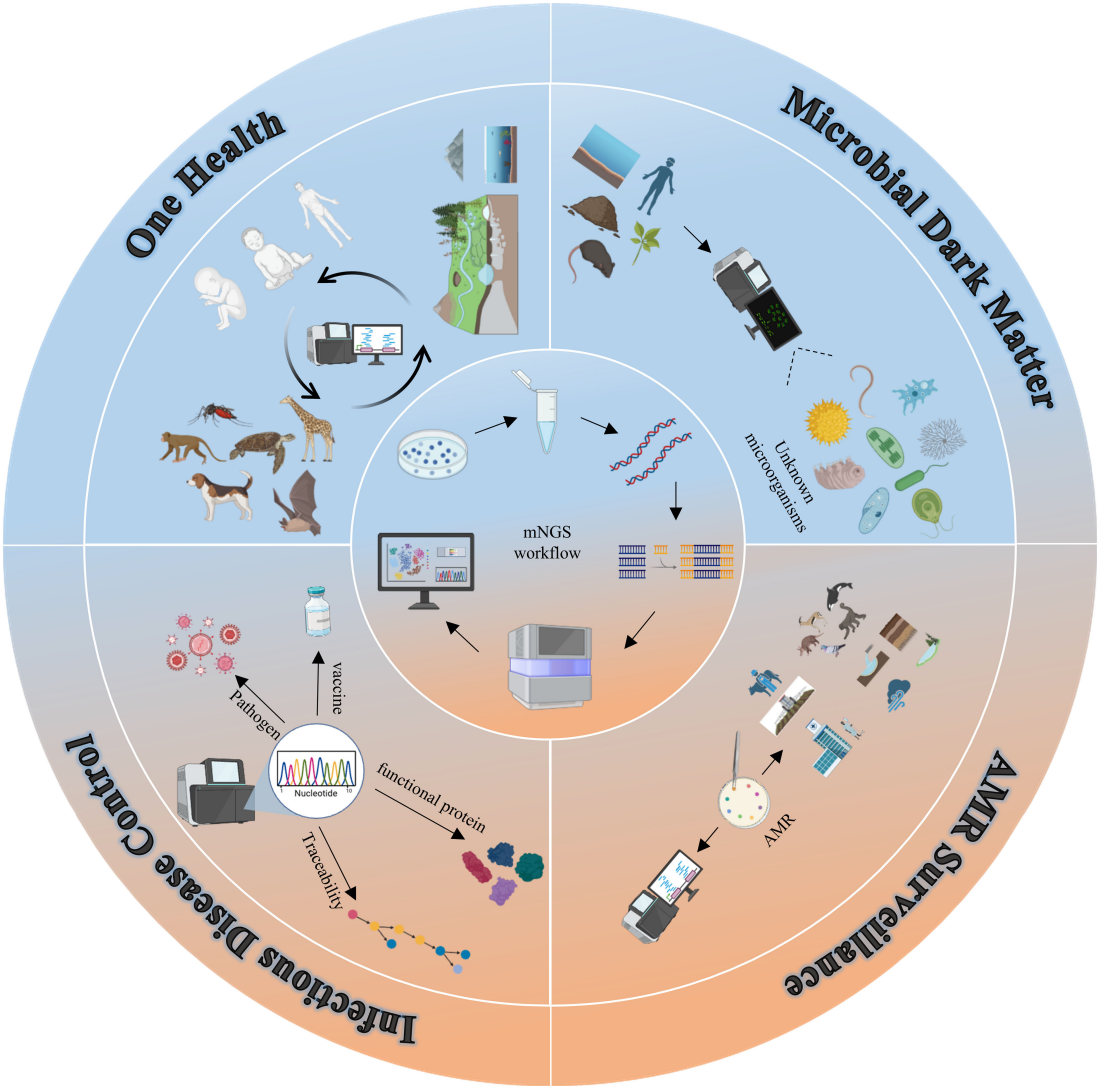
The core role of metagenomic next-generation sequencing in modern public health defense systems.

As a cornerstone of modern public health and microbial ecology (**Figure 2**), mNGS is poised to drive transformative advances across multiple fronts: proactive discovery of emerging pathogens, high-resolution outbreak tracing, surveillance of antimicrobial resistance across human–animal–environment interfaces, and integrated One Health monitoring. Its continued evolution promises to strengthen global capacity for early threat detection and evidence-based intervention.

Realizing this target, however, requires addressing challenges beyond the technical domain. We urgently call for three coordinated actions: (i) establishing global standardized workflows to ensure reproducibility and interoperability; (ii) strengthening international collaboration networks that enable real-time data sharing while respecting data sovereignty and ethics; and (iii) investing in computational infrastructure and bioinformatics capacity, particularly in resource-limited settings, to ensure equitable access. Advancing these priorities will reshape public health surveillance and build a more resilient global health system for future generations.
